# Differential predictability for high-risk plaque characteristics between fractional flow reserve and instantaneous wave-free ratio

**DOI:** 10.1038/s41598-023-43352-y

**Published:** 2023-09-25

**Authors:** Joo Myung Lee, Doosup Shin, Seung Hun Lee, Ki Hong Choi, Sung Mok Kim, Eun Ju Chun, Kwan Yong Lee, Doyeon Hwang, Sung Gyun Ahn, Adam J. Brown, Hernán Mejía-Rentería, Adrien Lefieux, David Molony, Kiyuk Chang, Tsunekazu Kakuta, Javier Escaned, Habib Samady

**Affiliations:** 1 Division of Cardiology, Department of Internal Medicine, Heart Vascular Stroke Institute, Samsung Medical Center, Sungkyunkwan University School of Medicine, 81 Irwon-ro, Gangnam-gu, Seoul, Republic of Korea; 2 Division of Cardiology, Duke University Medical Center, Durham, NC USA; 3 Department of Internal Medicine and Cardiovascular Center, Chonnam National University Hospital, Gwangju, Republic of Korea; 4 Department of Radiology, Cardiovascular Imaging Center, Heart Vascular Stroke Institute, Samsung Medical Center, Sungkyunkwan University School of Medicine, Seoul, Republic of Korea; 5 Department of Radiology, Seoul National University Bundang Hospital, Seongnam, Republic of Korea; 6 Cardiovascular Center and Cardiology Division, Seoul St. Mary’s Hospital, The Catholic University of Korea, Seoul, Republic of Korea; 7 Department of Internal Medicine and Cardiovascular Center, Seoul National University Hospital, Seoul, Republic of Korea; 8 Division of Cardiology, Department of Internal Medicine, Yonsei University Wonju College of Medicine, Wonju Severance Christian Hospital, Wonju, Republic of Korea; 9 Monash Cardiovascular Research Centre, Monash University and MonashHeart, Monash Health, Clayton, VIC Australia; 10 Hospital Clinico San Carlos IDISSC, Complutense University of Madrid, Madrid, Spain; 11 Covanos, Inc., Atlanta, GA USA; 12 Andreas Gruentzig Cardiovascular Center, Department of Medicine, Division of Cardiology, Emory University School of Medicine, Atlanta, GA USA; 13 Georgia Heart and Vascular Institute, Northeast Georgia Health System, 200 South Enota Drive, Suite 430, Gainesville, GA 30501 USA; 14 Division of Cardiovascular Medicine, Tsuchiura Kyodo General Hospital, Tsuchiura, Ibaraki Japan

**Keywords:** Interventional cardiology, Coronary artery disease and stable angina, Atherosclerosis, Cardiology

## Abstract

To evaluate the differential associations of high-risk plaque characteristics (HRPC) with resting or hyperemic physiologic indexes (instantaneous wave-free ratio [iFR] or fractional flow reserve [FFR]), a total of 214 vessels from 127 patients with stable angina or acute coronary syndrome who underwent coronary computed tomography angiography (CCTA) and invasive physiologic assessment were investigated. HPRC were classified into quantitative (minimal luminal area < 4 mm^2^ or plaque burden ≥ 70%) and qualitative features (low attenuation plaque, positive remodeling, napkin ring sign, or spotty calcification). Vessels with FFR ≤ 0.80 or iFR ≤ 0.89 had significantly higher proportions of HRPC than those with FFR > 0.80 or iFR > 0.89, respectively. FFR was independently associated with both quantitative and qualitative HRPC, but iFR was only associated with quantitative HRPC. Both FFR and iFR were significantly associated with the presence of ≥ 3 HRPC, and FFR demonstrated higher discrimination ability than iFR (AUC 0.703 vs. 0.648, *P* = 0.045), which was predominantly driven by greater discriminating ability of FFR for quantitative HRPC (AUC 0.832 vs. 0.744, *P* = 0.005). In conclusion, both FFR and iFR were significantly associated with CCTA-derived HRPC. Compared with iFR, however, FFR was independently associated with the presence of qualitative HRPC and showed a higher predictive ability for the presence of ≥ 3 HRPC.

## Introduction

Since a fundamental goal of coronary revascularization is relieving myocardial ischemia and symptoms caused by flow-limiting epicardial coronary stenosis, fractional flow reserve (FFR) and instantaneous wave free ratio (iFR) are currently used to guide revascularization^1^. However, another important goal of evaluating patients with coronary artery disease is to identify, and ultimately reduce, the risk of future cardiac events such as acute coronary syndrome (ACS) and sudden cardiac death. Since postmortem studies provided insights into morphological features of high-risk vulnerable plaque as the major cause of ACS and sudden cardiac death^2,3^, several imaging modalities such as intravascular imaging or coronary computed tomography angiography (CCTA) have identified high-risk plaque characteristics (HRPC) that predict future ACS and cardiac death^4–7^.

Recent studies linking the functional significance of a coronary stenosis as assessed by FFR to HRPC observed on CCTA^3,8–12^ may, in part, explain the observed reduction in spontaneous myocardial infarction (MI) in patients undergoing FFR-guided percutaneous coronary intervention (PCI)^13,14^. Unlike for FFR, there are limited data describing a relationship between iFR and HRPC^10^. Yet, there are reasons to consider that such a relationship might be different. On one hand, stenosis assessment with FFR is performed under conditions of maximal hyperemic flow, while iFR assesses stenosis severity using resting or non-hyperemic flow. On the other, iFR correlates better with estimates of microcirculatory flow modulation like coronary flow reserve than FFR^15^. Both maximal hyperemic flow and autoregulation affect epicardial vessel biology through mechanisms like modification of wall shear stress^16^, thus play a role in atheromatous plaque destabilization.

To address this void of knowledge, in the present study we investigated the association between CCTA-derived HRPC and invasively measured FFR and iFR. We further examined whether any differences in the predictive ability of the invasive physiologic indexes were related to quantitative or qualitative HRPC.

## Methods

### Study population

The current study was retrospectively conducted based on the international, multicenter registry which enrolled 361 vessels from 237 patients who underwent CCTA followed by invasive coronary angiography with physiologic assessments for stable angina or ACS at tertiary medical centers in Korea, Japan, Australia, and Spain, where CCTA and physiologic assessments have been routinely done in daily practice for the evaluation of coronary artery disease. The registry was established to examine diagnostic accuracy of CCTA-derived FFR values calculated based on the novel computational fluid dynamics method, compared with invasively measured physiologic indexes. Among them, 86 vessels without both FFR and iFR measurements were excluded. Then, vessels with poor CCTA image quality (n = 24), insufficient CCTA images for core laboratory analysis (n = 14), severe image artifact (n = 14), or prior stents or coronary artery bypass graft (n = 9) were excluded. Following these exclusions, a total of 214 vessels from 127 patients were analyzed in the current study. The study protocol was approved and the requirement for informed consent of the individual patients was waived by the Institutional Review Board at Samsung Medical Center, South Korea due to retrospective nature of the study. The study protocol was in accordance with the Declaration of Helsinki.

### Coronary computed tomography angiography and high-risk plaque characteristics

All patients underwent CCTA with 64 or higher detector row scanner platforms. In accordance with the Society of Cardiovascular Computed Tomography guidelines^17^, the CCTA images were analyzed at a core laboratory (Seoul National University Bundang Hospital, Seongnam, Korea) in a blinded fashion. Plaque in the most severe stenosis within the target vessel was selected for per-vessel analyses. Quantitative features of target vessels and lesions were analyzed, including minimal luminal area (MLA), plaque burden in MLA segment, total aggregated plaque volume (TAPV), and percent TAPV (TAPV/total vessel volume X 100). Based on previous invasive imaging^4,5,18^ and CCTA-based studies ^6,8,18–20^, MLA < 4 mm^2^ and plaque burden ≥ 70% were considered to be quantitative HRPC. Qualitative features of plaques were identified based on previous studies which reported definitions and predictive values of 4 qualitative HRPC for subsequent adverse clinical outcomes^6,8,18–20^: low attenuation plaque, positive remodeling, napkin ring sign, and spotty calcification. Briefly, plaque density was assessed semi-automatically using a dedicated cardiac workstation (Intellispace Portal, Philips Healthcare, Cleveland, OH, USA)^19^. Low attenuation plaque was defined as a plaque with an average density ≤ 30 Hounsfield units (HU) from 3 random region-of-interest in noncalcified portion of the plaque^8^. Positive remodeling was defined as a remodeling index ≥ 1.1, a ratio of maximal diameters between lesion and proximal reference vessel^3,6^. Napkin ring sign was characterized as a low attenuating plaque core surrounded by a ring-like area of higher attenuation^8,20^. Spotty calcification was defined as an intralesional calcification with an average density > 130 HU, diameter < 3 mm in any direction, length < 1.5 times the vessel diameter, and width of the calcification less than two-thirds of the vessel diameter^3,8^.

Consequently, HRPC was defined if there was any quantitative (MLA < 4 mm^2^ or plaque burden ≥ 70%) or qualitative feature (low attenuation plaque, positive remodeling, napkin ring sign, or spotty calcification) noted, and the presence of ≥ 3 HRPC from any combination of quantitative and/or qualitative HRPC features was considered to be significant, since it was found to be independently associated with adverse outcomes^8^.

### Angiographic analysis and quantitative coronary angiography

Coronary angiography was performed using standard techniques and median interval between CCTA and coronary angiogram was 15.5 days (interquartile range: 5–30 days). Angiographic views were obtained after administration of intracoronary nitrate (100–200 μg). All angiograms were analyzed at a core laboratory in a blinded fashion. Quantitative coronary angiography was performed in optimal projections with validated software (CAAS II, Pie Medical System, Maastricht, The Netherlands). Minimal lumen diameter, reference vessel size, percent diameter stenosis (%DS), and lesion length were measured.

### Invasive physiologic assessments

All coronary physiologic indexes were measured after diagnostic angiography. After a pressure wire sensor was zeroed and equalized to the aortic pressure, it was positioned at the distal segment of a target vessel. Intracoronary nitrate (100–200 µg) was administered before each set of physiologic measurements. For the physiologic assessment, a ratio between proximal (Pa) and distal coronary pressure (Pd) was obtained during resting and maximal hyperemia. iFR was calculated as a resting Pd/Pa measured during the wave-free period of diastole^21^. If iFR was not obtained during the procedure, it was calculated using resting pressure tracings in post-hoc manner (15.4%; N = 33/214), as previously described^22^. FFR was calculated as the lowest average of mean Pd/Pa from 3 consecutive beats during maximal hyperemia. All coronary physiologic measurements were analyzed at a core laboratory in a blinded fashion.

### Statistical analysis

Data were analyzed on a per-vessel basis and generalized estimating equation (GEE) models with independent correlation structures were used to adjust for intrasubject variability among vessels from the same patient and participating center^8^. CCTA-measured characteristics of target vessels and HRPC were compared between groups according to FFR or iFR with 0.80 and 0.89 as cutoff values, respectively. In addition, the CCTA-derived characteristic and number of HRPC (0, 1, 2, and ≥ 3) were compared among 4 groups classified by FFR and iFR values. Associations between physiologic indexes and the presence of ≥ 3 HRPC were evaluated using multivariable GEE model with logistic regression. Covariables were age, sex, diabetes mellitus, presentation with ACS, current smoking, dyslipidemia, and plaque in proximal segment of a target vessel. To investigate independent associations, presence of quantitative HRPC was further adjusted when looking at the associations between physiologic indexes and qualitative HRPC, and vice versa. In addition, the associations of physiologic indexes with probability of the presence of ≥ 3 HRPC were graphically presented with penalized splines with a degree of freedom of 3. Discrimination abilities of FFR and iFR for the presence of ≥ 3 HRPC as well as quantitative and qualitative HRPC were compared using receiver operating characteristic (ROC) curve and area under the curve (AUC). All probability values were 2-sided and *p*-values < 0.05 were considered statistically significant.

## Results

### Characteristics of patients and target lesions

**Figure 1:**
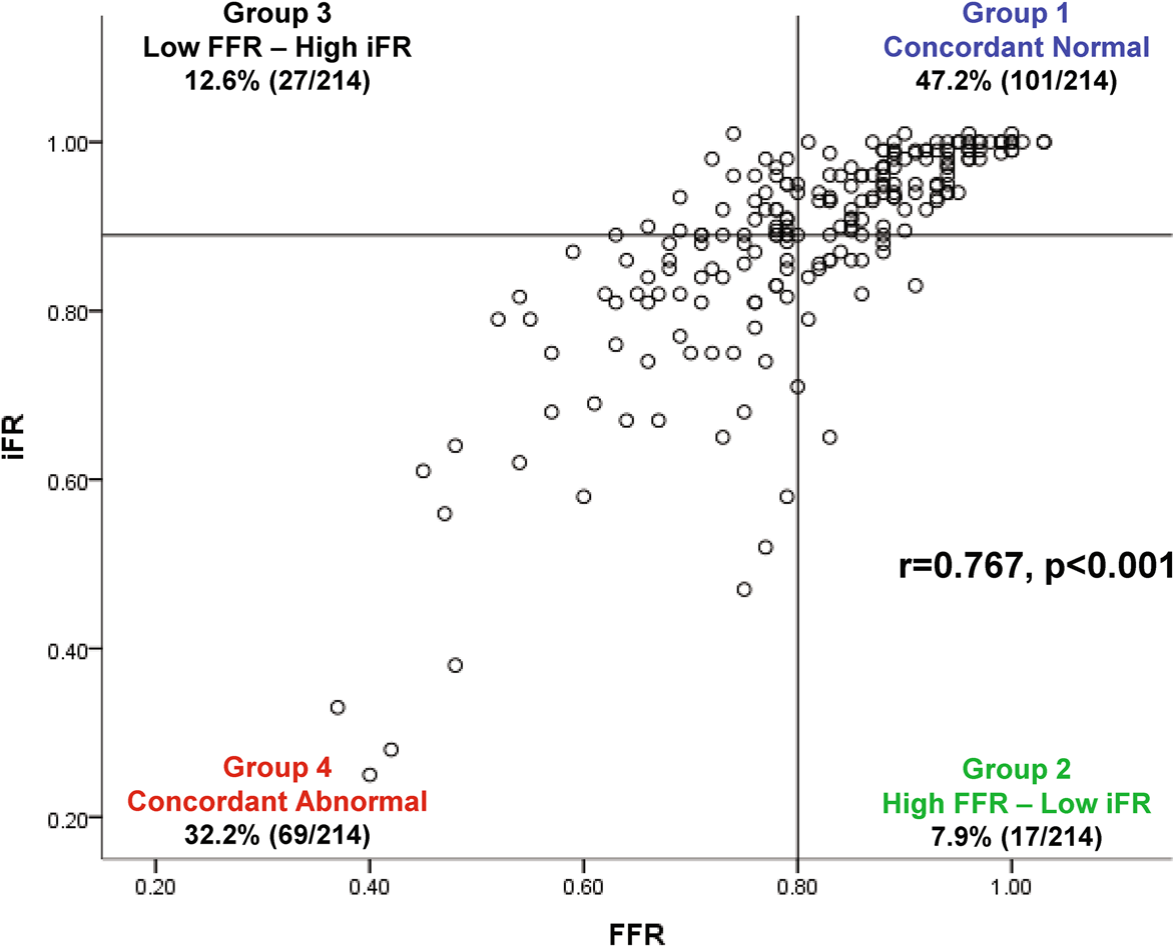
Relationship between FFR and iFR. Scatter plot between FFR and iFR is presented. *Abbreviations* FFR, fractional flow reserve; iFR, instantaneous wave-free ratio.

Tables 1 and 2 show general characteristics of the study population and target vessels, respectively. In brief, mean age was 64.9 ± 10.9 years and 74.0% were men. Most patients (88.2%) presented with stable angina. Mean FFR was 0.81 ± 0.13 and 44.9% had positive FFR ≤ 0.80. Mean iFR was 0.88 ± 0.13 and 40.2% had positive iFR ≤ 0.89. There was a significant correlation between FFR and iFR (r = 0.767, *P* < 0.001) and discordance between FFR- and iFR-based classifications occurred in 20.6% (Fig. 1).

**Table 1:** General characteristics of patients.

	N = 127
General characteristics	
Age, years	64.9 ± 10.9
Male	94 (74.0%)
CCTA-CAG interval, days	15 (4–30)
Cardiovascular risk factors	
Hypertension	91 (71.7%)
Diabetes mellitus	59 (46.5%)
Hypercholesterolemia	76 (59.8%)
Chronic kidney disease	7 (5.5%)
Current smoker	69 (54.3%)
Previous MI	5 (3.9%)
Previous PCI	13 (10.2%)
Clinical presentations	
Stable angina	112 (88.2%)
Unstable angina	10 (7.9%)
NSTEMI	5 (3.9%)
Agatston Calcium score	220.3 (101.3–486.6)

Values are n (%), mean ± standard deviation, or median (Q1–Q3).

*CAG* Coronary angiography, *CCTA* Coronary computed tomography angiography, *MI* Myocardial infarction, *NSTEMI* Non-ST-segment elevation myocardial infarction, *PCI* Percutaneous coronary intervention.

**Table 2:** Comparison of general characteristics and CCTA-derived characteristics of target vessels.

	Total	FFR > 0.80	FFR ≤ 0.80	*P* value^†^	iFR > 0.89	iFR ≤ 0.89	*P* value^‡^
Angiographic parameters	214	118 (55.1%)	96 (44.9%)		128 (59.8%)	86 (40.2%)	
Target vessel location				< 0.001			< 0.001
Left anterior descending artery	109 (50.9%)	42 (35.6%)	67 (69.8%)		41 (32.0%)	68 (79.1%)	
Left circumflex artery	47 (22.0%)	35 (29.7%)	12 (12.5%)		33 (25.8%)	14 (16.3%)	
Right coronary artery	58 (27.1%)	41 (34.7%)	17 (17.7%)		54 (42.2%)	4 (4.7%)	
Instantaneous wave-free ratio	0.88 ± 0.13	0.95 ± 0.06	0.81 ± 0.15	< 0.001	0.96 ± 0.03	0.77 ± 0.14	< 0.001
Fractional flow reserve	0.81 ± 0.13	0.90 ± 0.06	0.70 ± 0.10	< 0.001	0.88 ± 0.08	0.71 ± 0.12	< 0.001
Quantitative coronary angiography							
Reference diameter, mm	3.3 ± 0.8	3.5 ± 0.9	3.2 ± 0.6	0.166	3.5 ± 0.9	3.1 ± 0.7	0.019
Minimum lumen diameter, mm	1.7 ± 0.6	2.0 ± 0.5	1.5 ± 0.5	< 0.001	2.0 ± 0.5	1.5 ± 0.5	< 0.001
Diameter stenosis, %	47.6 ± 14.8	42.0 ± 14.4	54.2 ± 12.4	< 0.001	42.6 ± 14.3	53.5 ± 13.2	0.001
Lesion length, mm	16.5 ± 8.3	14.8 ± 7.8	18.4 ± 8.5	0.065	15.2 ± 8.0	18.0 ± 8.5	0.149
Computed tomography parameters	214	118 (55.1%)	96 (44.9%)		128 (59.8%)	86 (40.2%)	
Quantitative parameters							
MLA, mm^2^	2.3 ± 1.5	2.7 ± 1.7	1.8 ± 1.2	< 0.001	2.6 ± 1.7	1.8 ± 1.1	< 0.001
MLA < 4 mm^2^	163 (76.2%)	77 (65.3%)	86 (89.6%)	< 0.001	85 (66.4%)	78 (90.7%)	< 0.001
Plaque burden, %	77.8 ± 12.7	74.3 ± 13.2	81.3 ± 11.1	< 0.001	75.5 ± 13.4	80.6 ± 11.1	0.006
Plaque burden ≥ 70%	144 (67.3%)	69 (58.5%)	75 (78.1%)	0.002	77 (60.2%)	67 (77.9%)	0.007
Diameter stenosis, %	43.2 ± 24.6	30.7 ± 20.3	58.6 ± 20.5	< 0.001	33.4 ± 22.2	57.8 ± 20.6	< 0.001
Remodeling index	1.05 ± 0.09	1.04 ± 0.07	1.06 ± 0.10	0.077	1.04 ± 0.08	1.06 ± 0.09	0.188
Low attenuation plaque volume, mm^3^	4.9 ± 11.2	3.2 ± 6.3	6.7 ± 14.3	0.037	3.3 ± 5.9	7.0 ± 15.2	0.027
Percent TAPV, %	59.8 ± 11.5	57.1 ± 12.2	62.6 ± 10.2	0.001	58.1 ± 11.4	62.0 ± 11.4	0.022
Qualitative parameters							
Low attenuation plaque	18 (8.4%)	4 (3.4%)	14 (14.6%)	0.003	7 (5.5%)	11 (12.8%)	0.058
Positive remodeling	43 (20.1%)	13 (11.0%)	30 (31.3%)	< 0.001	18 (14.1%)	25 (29.1%)	0.007
Napkin ring sign	19 (8.9%)	5 (4.2%)	14 (14.6%)	0.008	5 (3.9%)	14 (16.3%)	0.002
Spotty calcification	34 (15.9%)	15 (12.7%)	19 (19.8%)	0.159	20 (15.6%)	14 (16.3%)	0.898
Number of high-risk plaque characteristics ≥ 3*	65 (30.4%)	25 (21.2%)	40 (41.7%)	0.001	31 (24.2%)	34 (39.5%)	0.017

Values are mean ± standard deviation or n (%). Generalized estimating equation model or maximum likelihood χ^2^ tests were used for overall and between groups comparison in per-vessel analysis.

*CCTA* Coronary computed tomography angiography, *FFR* Fractional flow reserve, *iFR* Instantaneous wave-free ratio, *MLA* Minimal lumen area, *TAPV* Total aggregated plaque volume.

^†^*P* values for the comparison of variables between high and low FFR groups. ^‡^*P* values for the comparison of variables between high and low iFR groups.

*High risk plaque characteristics: 1) Plaque burden ≥ 70%; 2) MLA < 4mm^2^;3) Positive remodeling; 4) Low attenuation plaque; 5) Napkin ring sign; 6) Spotty calcification.

### CCTA-derived characteristics and physiologic indexes

Table 2 demonstrates CCTA-derived characteristics of target vessels and lesions according to FFR and iFR. Compared to the lesions with negative FFR > 0.80 or iFR > 0.89, those with positive FFR ≤ 0.80 or iFR ≤ 0.89 had significantly higher incidence of quantitative and qualitative HRPC as well as lesions with ≥ 3 HRPC, respectively (FFR: 21.2% vs. 41.7%, respectively, *P* = 0.001; iFR: 24.2% vs. 39.5%, respectively, *P* = 0.017). In addition, there were significant differences in FFR or iFR values according to the number of HRPC (*P* < 0.001 and *P* = 0.001, respectively; Supplementary Fig. 1), and FFR and iFR values were inversely associated with the number of HRPC (*P* < 0.001 for both; Supplementary Fig. 2).

### CCTA-derived characteristics according to agreement between FFR and iFR

**Figure 2:**
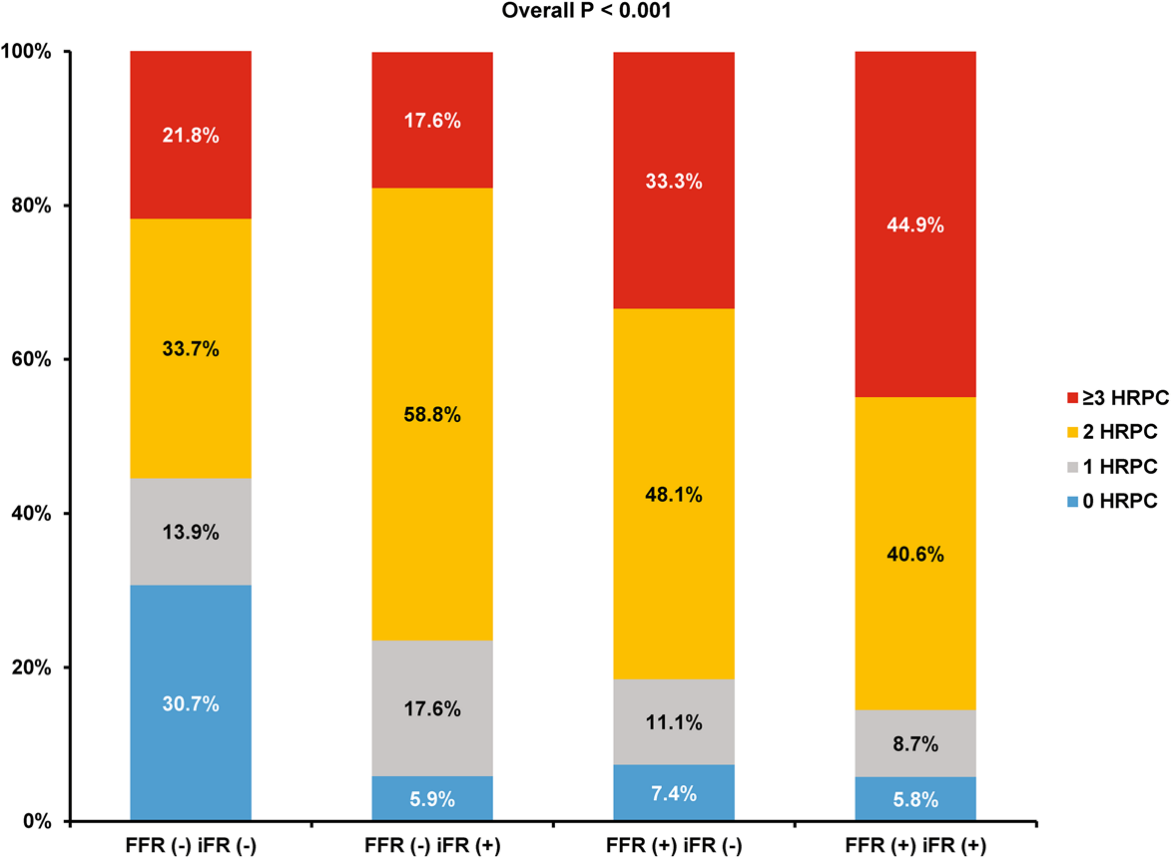
Proportion of High-Risk Plaque Characteristics Among 4 Groups, Classified by FFR and iFR. Proportions of number of HRPC (0, 1, 2, and ≥ 3) are compared according to the 4 groups classified by FFR and iFR with cutoff values of 0.80 and 0.89, respectively. *Abbreviations* HRPC, high-risk plaque characteristics; other abbreviations as in Fig. 1.

Table 3 shows CCTA-derived characteristics of target vessels and lesions according to the 4 groups classified by FFR and iFR. There was significant difference in the proportion of lesions with ≥ 3 HRPC among the 4 groups. Group 1 with concordantly negative FFR and iFR had the lowest proportion of lesions with ≥ 3 HRPC and group 4 with concordantly positive FFR and iFR had the highest proportion of lesions with ≥ 3 HRPC (overall *P* < 0.001). Furthermore, group 4 showed a consistently higher number of lesions with quantitative or qualitative HRPC than the other groups. Among the discordant groups, group 3 with positive FFR but negative iFR showed a numerically higher proportion of lesions with ≥ 3 HRPC than group 2 with negative FFR but positive iFR (Table 3 and Fig. 2).

**Table 3:** Comparison of CCTA-derived characteristics according to 4 groups classified by FFR and iFR.

	Group 1 FFR (−) / iFR (−)	Group 2 FFR (−) / iFR ( +)	Group 3 FFR ( +) / iFR (−)	Group 4 FFR ( +) / iFR ( +)	*P* value
Computed tomography parameters	101 (47.2%)	17 (7.9%)	27 (12.6%)	69 (32.2%)	
Quantitative parameters					
MLA, mm^2^	2.9 ± 1.8	2.0 ± 1.1	1.9 ± 1.3	1.7 ± 1.1	< 0.001
MLA < 4 mm^2^	62 (61.4%)	15 (88.2%)	23 (85.2%)	63 (91.3%)	< 0.001
Plaque burden, %	73.5 ± 13.3	78.4 ± 12.3	81.7 ± 12.1	81.1 ± 10.8	0.001
Plaque burden ≥ 70%	55 (54.5%)	14 (82.4%)	22 (81.5%)	53 (76.8%)	0.002
Diameter stenosis, %	29.3 ± 20.4	39.3 ± 18.1	48.9 ± 22.1	62.4 ± 18.6	< 0.001
Remodeling index	1.03 ± 0.08	1.05 ± 0.06	1.06 ± 0.09	1.06 ± 0.10	0.338
Low attenuation plaque volume, mm^3^	3.0 ± 5.6	4.4 ± 8.9	4.3 ± 6.8	7.6 ± 16.3	0.102
Percent TAPV, %	56.9 ± 11.8	58.5 ± 14.4	62.0 ± 9.3	62.9 ± 10.5	0.011
Qualitative parameters					
Low attenuation plaque	4 (4.0%)	0 (0.0%)	3 (11.1%)	11 (15.9%)	0.023
Positive remodeling	11 (10.9%)	2 (11.8%)	7 (25.9%)	23 (33.3%)	0.003
Napkin ring sign	3 (3.0%)	2 (11.8%)	2 (7.4%)	12 (17.4%)	0.013
Spotty calcification	15 (14.9%)	0 (0.0%)	5 (18.5%)	14 (20.3%)	0.218
Number of high-risk plaque characteristics ≥ 3*	22 (21.8%)	3 (17.7%)	9 (33.3%)	31 (44.9%)	0.008

Values are mean ± standard deviation, or n (%). Generalized estimating equation model or maximum likelihood χ^2^ tests were used for overall and between groups comparison in per-vessel analysis.

*CCTA* Coronary computed tomography angiography, *FFR* Fractional flow reserve, *iFR* Instantaneous wave-free ratio, *MLA* Minimal lumen area, *TAPV* Total aggregated plaque volume.

*High risk plaque characteristics: 1) Plaque burden ≥ 70% 2) MLA < 4mm^2^ 3) Positive remodeling 4) Low attenuation plaque 5) Napkin ring sign 6) Spotty calcification.

### Associations between CCTA-derived HRPC and physiologic indexes

**Figure 3:**
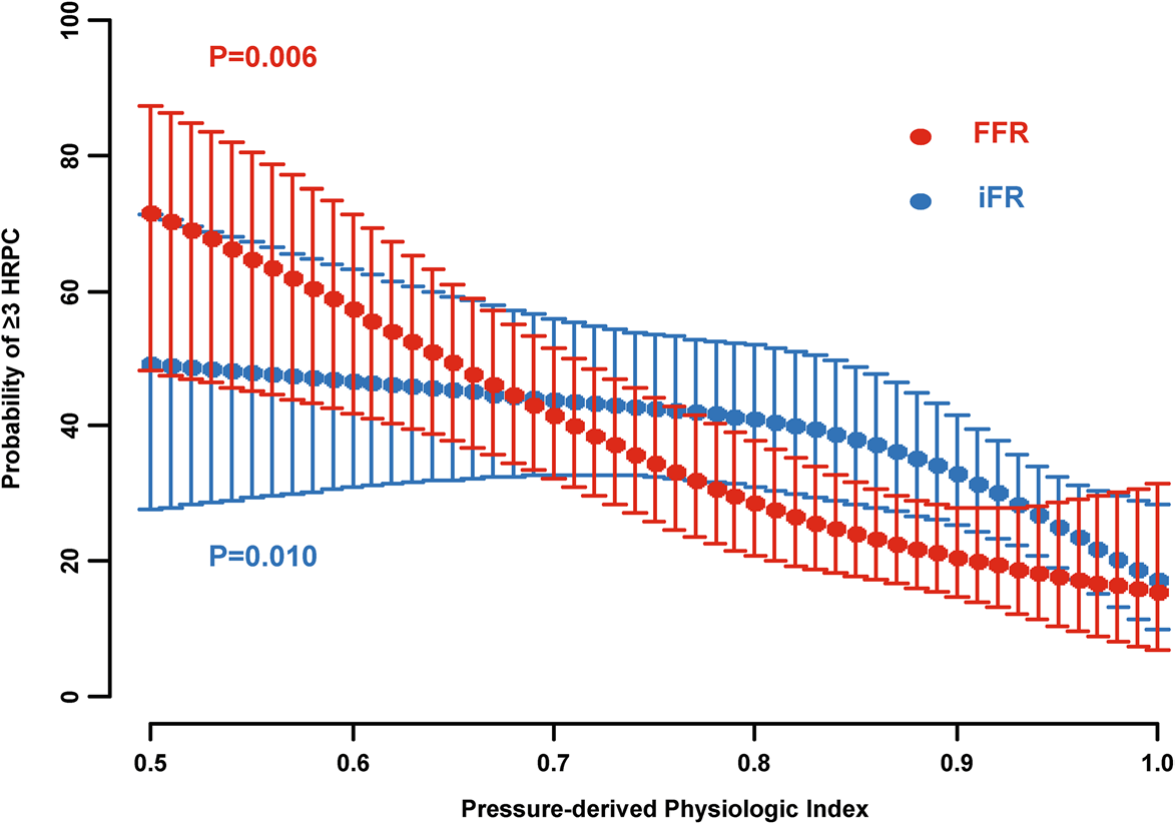
Association of FFR or iFR with Probability of ≥ 3 High-Risk Plaque Characteristics. Probabilities of the presence of ≥ 3 HRPC are plotted according to FFR and iFR values. Abbreviations as in Fig. 2.

After adjusting for various patient- and vessel-related characteristics including age, sex, diabetes mellitus, presentation with ACS, current smoking, dyslipidemia, and plaque in proximal segment of a target vessel, both FFR and iFR were significantly associated with the presence of ≥ 3 HRPC (per 0.01 decrease in FFR: OR 1.043, 95% CI 1.018–1.069, *P* = 0.001; per 0.01 decrease in iFR: OR 1.032, 95% CI 1.008–1.057, *P* = 0.008) (Table 4). After adjusting for quantitative HRPC, FFR was independently associated with qualitative HRPC, and vice versa. In contrast, iFR was independently associated with quantitative HRPC after adjusting for qualitative HRPC, but not with qualitative HRPC after adjusting for quantitative HRPC (Table 4). When the probability of ≥ 3 HRPC was plotted according to FFR or iFR values, both physiologic indexes were significantly associated with the probability of ≥ 3 HRPC (*P* = 0.006 and 0.010, respectively) (Fig. 3).

**Table 4:** Association of Patient/Vessel-related Characteristics with High-risk Plaque Characteristics.

Model with FFR	Overall HRPC ≥ 3		Quantitative HRPC*		Qualitative HRPC^†^	
	OR (95% CI)	*P* value	OR (95% CI)	*P* value	OR (95% CI)	*P* value
Age	1.00 (0.976–1.033)	0.764	1.041 (1.001–1.083)	0.043	0.997 (0.967–1.029)	0.857
Male	1.103 (0.455–2.671)	0.828	0.663 (0.198–2.227)	0.507	1.207 (0.492–2.961)	0.681
Diabetes mellitus	1.137 (0.616–2.096)	0.681	2.679 (1.101–6.515)	0.030	0.957 (0.508–1.805)	0.893
Acute coronary syndrome	0.762 (0.276–2.103)	0.600	0.787 (0.203–3.056)	0.730	0.862 (0.303–2.448)	0.780
Current smoking	1.934 (0.979–3.819)	0.057	1.572 (0.559–4.427)	0.391	1.862 (0.911–3.650)	0.090
Dyslipidemia	1.366 (0.723–2.578)	0.337	1.147 (0.465–2.832)	0.766	1.303 (0.681–2.498)	0.424
Plaque in proximal segment	1.414 (0.730–2.741)	0.305	1.757 (0.730–4.224)	0.208	1.302 (0.658–2.574)	0.449
FFR, per 0.01 decrease	1.043 (1.018–1.069)	0.001	1.126 (1.065–1.192)	< 0.001	1.028 (1.001–1.055)	0.041

Model with iFR	Overall HRPC ≥ 3		Quantitative HRPC*		Qualitative HRPC^†^	
	OR (95% CI)	*P* value	OR (95% CI)	*P* value	OR (95% CI)	*P* value
Age	0.994 (0.966–1.022)	0.668	1.040 (1.001–1.079)	0.042	0.992 (0.963–1.023)	0.609
Male	1.341 (0.524–3.431)	0.541	0.720 (0.226–2.296)	0.579	1.263 (0.517–3.084)	0.608
Diabetes mellitus	1.630 (0.868–3.061)	0.128	2.604 (1.124–6.031)	0.025	0.874 (0.469–1.627)	0.670
Acute coronary syndrome	0.867 (0.303–2.476)	0.789	0.757 (0.217–2.641)	0.663	0.958 (0.343–2.680)	0.935
Current smoking	1.852 (0.918–3.738)	0.085	1.365 (0.522–3.568)	0.525	1.816 (0.909–3.627)	0.091
Dyslipidemia	1.264 (0.649–2.460)	0.491	1.283 (0.553–2.976)	0.562	1.344 (0.700–2.580)	0.374
Plaque in proximal segment	1.339 (0.685–2.617)	0.393	2.425 (1.080–5.448)	0.032	1.412 (0.722–2.761)	0.313
iFR, per 0.01 decrease	1.032 (1.008–1.057)	0.008	1.074 (1.020–1.130)	0.007	1.013 (0.989–1.037)	0.290

*CI* Confidence interval, *FFR* Fractional flow reserve, *HRPC* High-risk plaque characteristics, *iFR* Instantaneous wave-free ratio, *OR* Odds ratio.

*Multivariable model included age, sex, acute coronary syndrome, current smoking, diabetes mellitus, dyslipidemia, plaque in proximal segment, and the presence of qualitative HRPC.

^†^Multivariable model included age, sex, acute coronary syndrome, current smoking, diabetes mellitus, dyslipidemia, plaque in proximal segment, and the presence of quantitative HRPC.

### Discrimination ability of physiologic indexes for quantitative and qualitative HRPC

**Figure 4:**
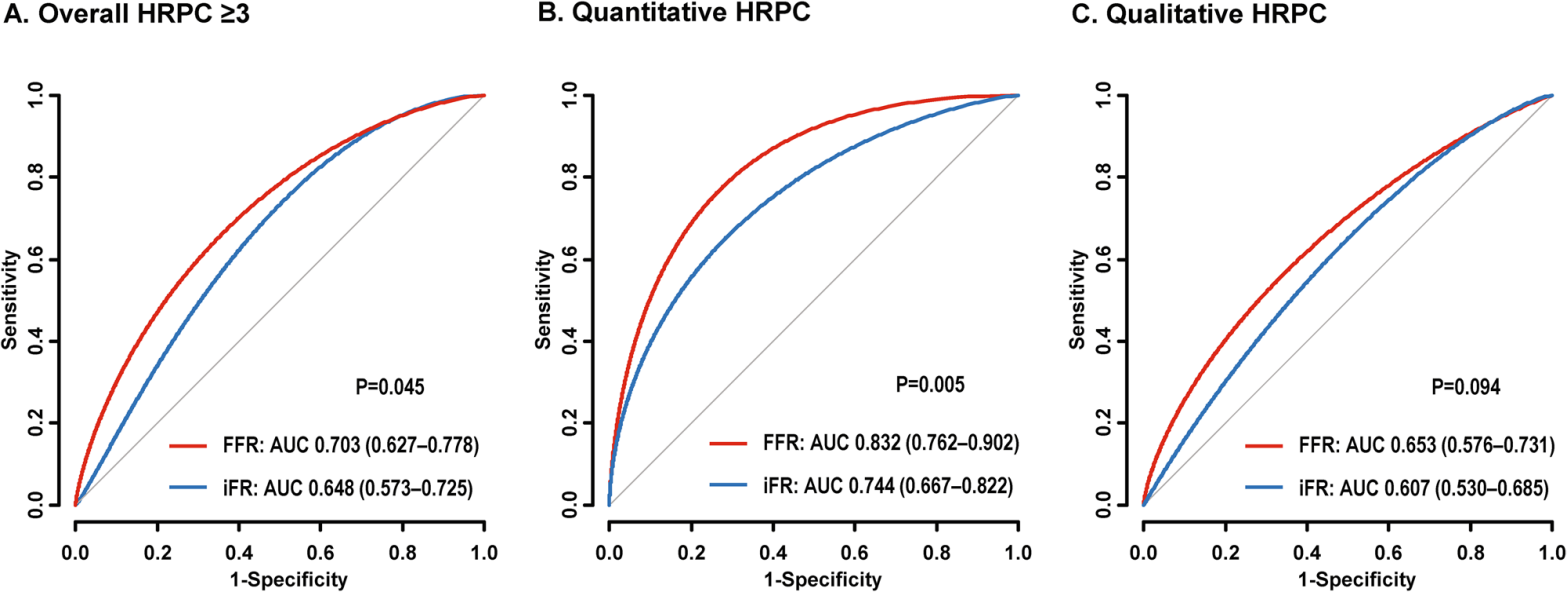
Comparison of Discrimination Ability for Quantitative or Qualitative High-Risk Plaque Characteristics between FFR and iFR. The receiver operating characteristic curves of FFR (in red) and iFR (in blue) to discriminate the presence of (**A**) overall HRPC ≥ 3, (**B**) quantitative HRPC, and (**C**) qualitative HRPC are presented. Quantitative HRPC included minimal luminal area < 4 mm^2^ and plaque burden ≥ 70%. Qualitative HRPC included low attenuation plaque, positive remodeling, napkin ring sign, and spotty calcification. *Abbreviations* AUC, area under the curve; other abbreviations as in Fig. 2.

To evaluate the discrimination ability of FFR and iFR for HRPC, ROC curves and AUC values were compared (Fig. 4). FFR showed a higher discrimination ability for the presence of ≥ 3 HRPC than iFR (AUC 0.703 vs. 0.648, respectively; *P* = 0.045). This difference was mainly driven by a higher discrimination ability of FFR for the presence of quantitative HRPC (AUC 0.832 vs. 0.744, respectively; *P* = 0.005). Among qualitative HRPC, FFR showed higher discrimination abilities to predict positive remodeling and napkin ring sign than iFR (*P* = 0.021 and 0.028, respectively), but no differences were seen for low attenuation plaque and spotty calcification (Supplementary Fig. 3).

## Discussion

The current study evaluated the association between CCTA-derived HRPC and invasively measured FFR and iFR. There were a number of observations. First, vessels with FFR ≤ 0.80 or iFR ≤ 0.89 had significantly higher number of both quantitative and qualitative HRPC. Second, both FFR and iFR were inversely associated with the number of HRPC. Even after adjusting for quantitative HRPC, FFR was independently associated with qualitative HRPC, but iFR was not. Third, although FFR showed a significantly higher predictability of the presence of ≥ 3 HRPC than iFR, the difference was mainly derived from a greater discrimination ability of FFR for the presence of quantitative HRPC (Central illustration).

### Quantitative and qualitative adverse plaque characteristics in coronary artery disease

Previous studies using intravascular ultrasound have identified 3 independent predictors of major adverse cardiac events (MACE): (1) small MLA ≤ 4.0 mm^2^; (2) large plaque burden ≥ 70%; and (3) virtual histology-defined thin-cap fibroatheroma (TCFA)^4,5,23^. Interestingly, no event occurred from untreated segments with a plaque burden < 40% in the Providing Regional Observations to Study Predictors of Events in the Coronary Tree (PROSPECT) study, suggesting an importance of quantitative plaque features^4^. Motoyama et al.^6^ reported higher event rates in patients with CCTA-derived qualitative HRPC (positive remodeling and/or low attenuation plaque), irrespective of the presence of significant stenosis ≥ 70%, representing an importance of qualitative plaque features. These results suggested that both quantitative and qualitative HRPC are important in risk stratification of patients for future adverse cardiac events.

### Association between coronary physiology and plaque vulnerability

The current standard decision-making for revascularization has been guided by invasive physiologic indexes as surrogates of coronary blood flow to define functional significant coronary stenosis. A recent meta-analysis demonstrated that FFR-guided PCI reduced the composite of cardiac death or MI compared with medical therapy alone^14^. In addition, the Fractional Flow Reserve Versus Angiography for Multivessel Evaluation (FAME) 2 trial demonstrated that FFR-guided strategy reduced the risk of spontaneous MI^13^. These results raised an important question as to how functionally significant lesions determined by physiologic indexes are related to high-risk plaques prone to cause ACS.

In fact, recent studies have shown that HRPC were closely related to FFR, and lesions with low FFR were likely to have more HRPC^3,8–12^. The current study also demonstrated that per-vessel FFR was inversely associated with the number of HRPC and the presence of quantitative and qualitative HRPC. There may be 2 potential mechanistic explanations for the relationship between FFR and HRPC. First, low FFR represents a high-pressure gradient across the lesion which can cause hemodynamic stress on a plaque, resulting in an adverse transformation of the plaque into more vulnerable form^8,19,24,25^. Among hemodynamic stress, wall shear stress (WSS) has been extensively studied. Park et al. demonstrated the significant association between lower FFR and higher WSS, which was also linked with the higher probability of CCTA-derived adverse plaque characteristics^19^. Second, the presence of HRPC could impair the local vasodilator capacity of the affected coronary segment via oxidative stress and inflammation, whereas that of an unaffected coronary segment is relatively preserved^9^. This differential vasodilatory capacity might induce a higher pressure gradient under maximal hyperemia and thereby decrease FFR.

### Hyperemic and non-hyperemic physiologic indexes and high-risk plaque characteristics

Based on the 2 representative randomized clinical trials, non-hyperemic pressure ratios (NHPRs) such as iFR have been used in clinical practice as alternatives to FFR^26,27^. However, unlike FFR and its previously described association with HRPC, there is limited evidence regarding the relationship between NHPRs and plaque vulnerability. A recent study by Driessen et al. reported that there might be a difference between hyperemic and non-hyperemic physiologic indexes in their relationship with CCTA-derived HRPC^10^. In their study, CCTA-derived qualitative HRPC, especially positive remodeling and spotty calcification, were independently associated with FFR, but not with iFR^10^. One potential explanation suggested by the authors was that a positively remodeled coronary segment containing lipid-rich plaques might not be able to dilate as much as an unaffected segment during maximal hyperemia, which could result in more obvious pressure gradient across the lesion during hyperemia than at rest^10^. Therefore, FFR could be more sensitive to the presence of HRPC, especially lipid-rich necrotic core and positive remodeling, than iFR. These results suggested that the effect of hyperemia on pressure-derived physiologic indexes might depend not only on quantitative stenosis severity but also on qualitative plaque characteristics^10^. The results of the current study also showed that FFR was independently associated with qualitative HRPC after adjusting for quantitative HRPC, but iFR was not. Furthermore, FFR showed a greater discrimination ability to predict positive remodeling than iFR, which could further support the above hypothesis.

Interestingly, the current study showed that FFR also had a higher discrimination ability for the presence of ≥ 3 HRPC than iFR, which was mainly driven by a higher sensitivity of FFR for the presence of quantitative HRPC. These findings may be related to the prior observation on FFR being more sensitive to anatomical stenosis severity than iFR, likely due to an increased flow separation and pressure loss across the stenosis during hyperemia than at rest^21^. Although the association between coronary physiology and plaque vulnerability would be far more complex, the current study provides evidence for the potential difference between hyperemic and non-hyperemic physiologic indexes, and their relationship with HRPC. Nonetheless, clinical significance of these differences is not clear given clinical trials showing comparable outcomes between FFR- and iFR-guided revascularization strategies^26,27^ and relatively low risk among lesions with discordant FFR and iFR values^28^. Rather, the current study should be regarded as an effort to enhance our understanding of complex interplay between hemodynamic and anatomic plaque characteristics.

In addition, it should be noted that the current study showed neither FFR nor iFR could be a perfect predictor for the presence of qualitative HRPC. These results support the complementary role of invasive physiologic indexes and CCTA-defined HRPC for risk stratification of patients with coronary artery disease. Considering the solid evidence for prognostic implication of invasive physiologic indexes, future studies investigating the incremental value of CCTA-derived HRPC over invasive physiologic indexes for predicting clinical outcomes are warranted. Furthermore, a clinical trial (The Preventive Coronary Intervention on Stenosis with Functionally Insignificant Vulnerable Plaque [PREVENT]; NCT02316886) is underway to test whether treating hemodynamically insignificant lesions with HRPC would provide any benefits, which may confirm or refute potential role of combining these complementary tools in the treatment of patients with coronary artery disease^29^.

The current study has several limitations. First, since it was conducted as a post hoc analysis, the influence of selection bias could not be fully excluded. Second, prognostic impact of the differential association between FFR and iFR for the presence of CCTA-derived HRPC is beyond the scope of the current study. Further study with clinical outcome is warranted. Third, other reference modalities to assess plaque characteristics, such as intravascular ultrasound or optical coherence tomography, were not systematically performed. Fourth, most patients included in the current study had stable angina. Therefore, our results could not be applied to non-culprit lesions in patients with ACS. Fifth, reproducibility of CCTA analysis was not specifically assessed in the current study, although it was done at the same core laboratory participated in the prior study^30^. Sixth, although physiologic disease pattern (focal vs. diffuse) can affect the discordance between FFR and iFR, it was not able to be considered in current study due to lack of pullback information.

In conclusion, both FFR and iFR were significantly associated with CCTA-derived HRPC. Compared with iFR, FFR was independently associated with the presence of qualitative HRPC and showed a higher predictive ability for the presence of ≥ 3 HRPC due primarily to a greater ability to predict quantitative HRPC. Further research is warranted to investigate the potentially incremental prognostic value of these observations.

### Supplementary Information

Supplementary Figures.

## Data Availability

The datasets generated during and/or analyzed during the current study are not publicly available but are available from the corresponding author on reasonable request.

## Abbreviations

CCTA: Coronary computed tomography angiography
FFR: Fractional flow reserve
HRPC: High risk plaque characteristics
iFR: Instantaneous wave-free ratio
NHPR: Non-hyperemic pressure ratio
