# Supplementary material for: Differential predictability for high-risk plaque characteristics between fractional flow reserve and instantaneous wave-free ratio
Source: Sci Rep. 2023 Sep 25;13:16005. doi: 10.1038/s41598-023-43352-y (PMC10520044; doi:10.1038/s41598-023-43352-y)
Supplement: Supplementary file 1 — Supplementary Figures. [file 41598_2023_43352_MOESM1_ESM.docx]

**Supplementary Appendix**

**Differential Predictability for High-Risk Plaque Characteristics Between Fractional Flow Reserve and Instantaneous Wave-Free Ratio**

Joo Myung Lee, MD, MPH, PhD, Doosup Shin, MD, Seung Hun Lee, MD, PhD, Ki Hong Choi, MD, Sung Mok Kim, MD, PhD, Eun Ju Chun, MD, PhD, Kwan Yong Lee, MD, PHD, Doyeon Hwang, MD, Sung Gyun Ahn, MD, PhD, Adam J. Brown, MD, PhD, Hernán Mejía-Rentería, MD, PhD, Adrien Lefieux, PhD, David Molony, PhD, Kiyuk Chang, MD, PHD, Tsunekazu Kakuta, MD, PhD, Javier Escaned, MD, PhD, Habib Samady, MD

**Table of Contents**

- **Supplementary Figures and Figure Legends**

**Supplementary Figures**

**Supplementary Figure 1. FFR and iFR According to Number of High-Risk Plaque Characteristics**


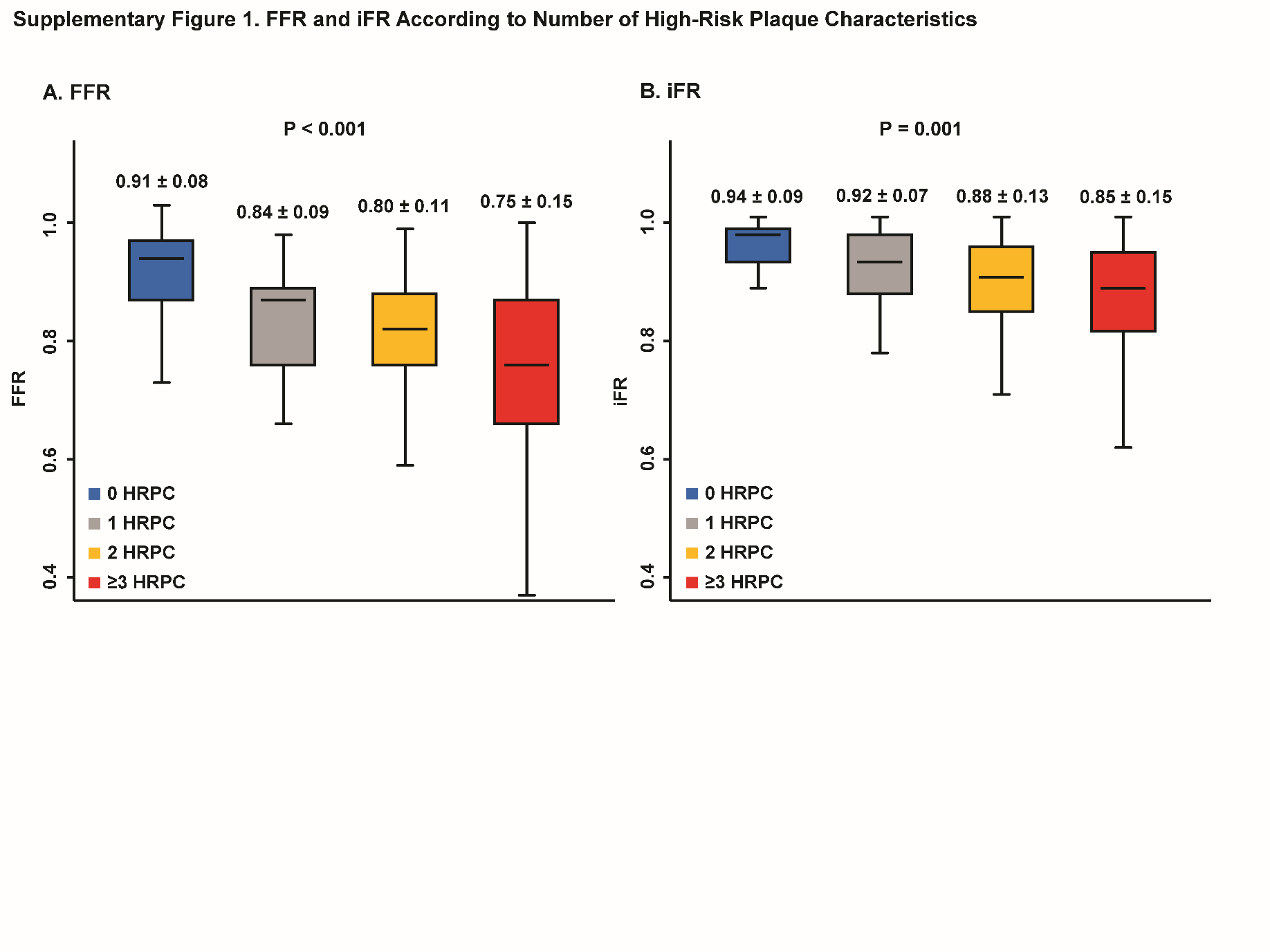


FFR and iFR values are compared according to number of HRPC (0, 1, 2, and ≥3). Values are means ± standard deviations. In box-and-whisker plots, horizontal lines indicate median values, boxes indicate the interquartile ranges, and whiskers indicate the minimum and maximum values.

Abbreviations: FFR, fractional flow reserve; HRPC, high-risk plaque characteristics; iFR, instantaneous wave-free ratio.

**Supplementary Figure 2. Number of High-Risk Plaque Characteristics According to FFR and iFR Quartiles**


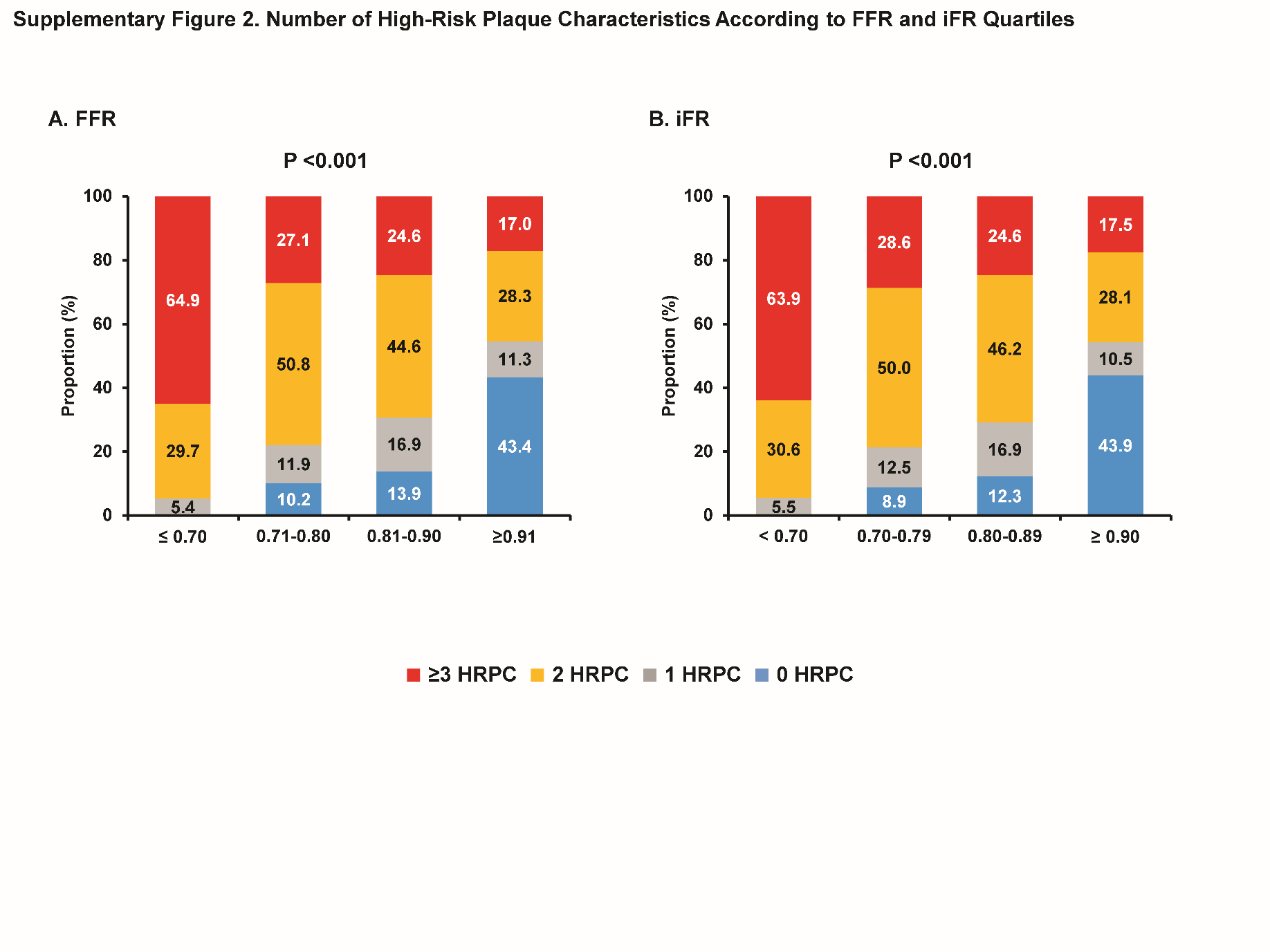


Proportions of number of HRPC (0, 1, 2, and ≥3) are compared according to the quartile values of (A) FFR and (B) iFR, respectively.

Abbreviations: FFR, fractional flow reserve; HRPC, high-risk plaque characteristics; iFR, instantaneous wave-free ratio.

**Supplementary Figure 3. Comparison of Discrimination Ability for Individual Components of Quantitative or Qualitative High-Risk Plaque Characteristics between FFR and iFR**


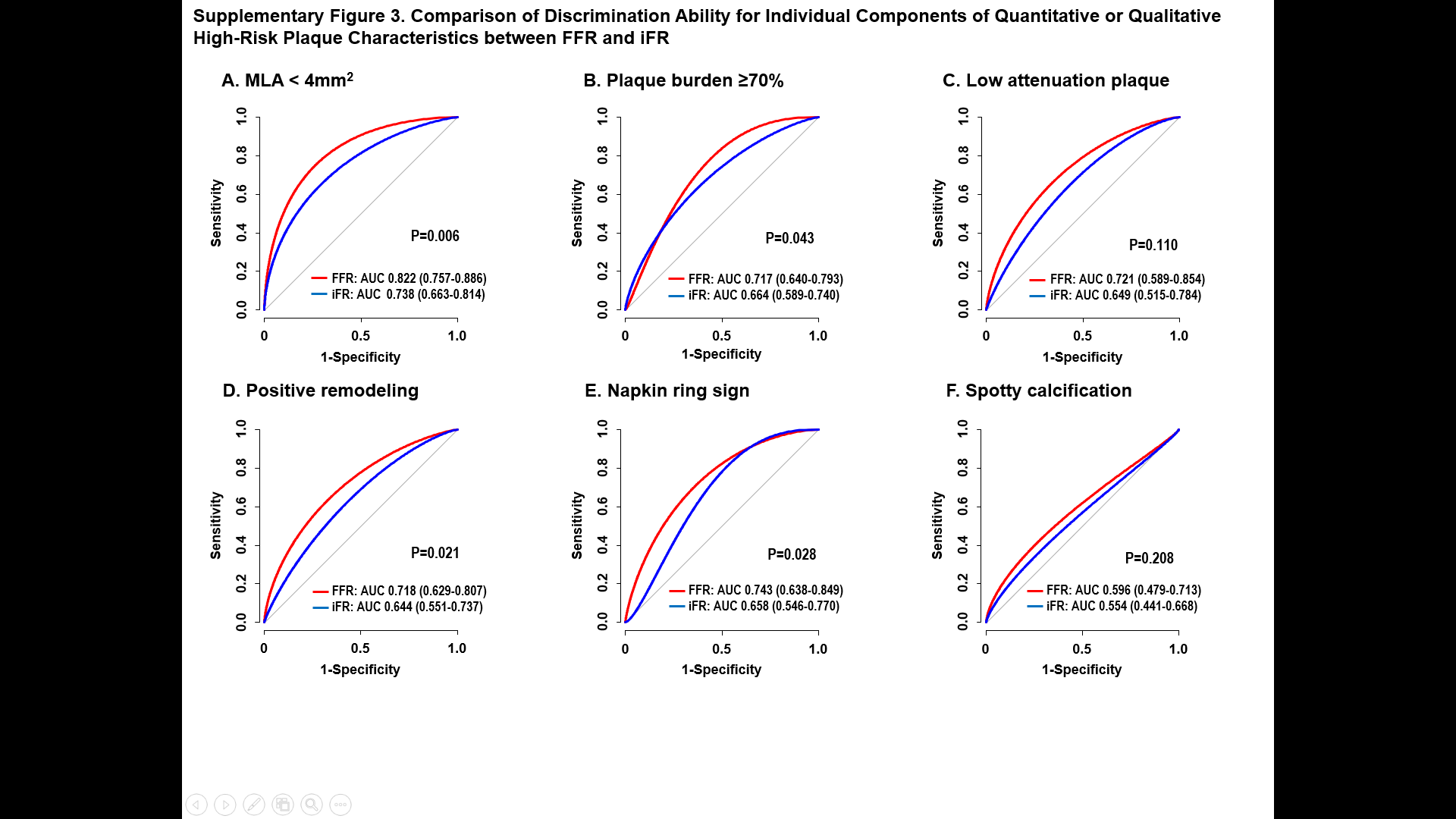


The receiver operating characteristic curves of FFR (in red) and iFR (in blue) to discriminate (A) MLA <4mm^2^, (B) plaque burden ≥70%, (C) low attenuation plaque, (D) positive remodeling, (E) napkin ring sign, and (F) spotty calcification are presented.

Abbreviations: AUC, area under the curve; FFR, fractional flow reserve; HRPC, high-risk plaque characteristics; iFR, instantaneous wave-free ratio; MLA, minimal lumen area.
